# An acute oral administration of Sildenafil in asthenozoospermic patients improves sperm motility after density gradient centrifugation

**DOI:** 10.5935/1518-0557.20200076

**Published:** 2021

**Authors:** Felipe A. Morales Martínez, Martha Merino Ruiz, Eddy E. Angulo Velarde, Otto H. Valdés Martínez, Sara P. Peña Lizola, Luis H. Sordia Hernández

**Affiliations:** 1 Centro Universitario de Medicina Reproductiva, Hospital Universitario “Dr. José Eleuterio González”, Universidad Autónoma de Nuevo León, Monterrey, Nuevo León, México

**Keywords:** Sildenafil, sperm motility, gradient centrifugation, PDE5

## Abstract

**Objective:**

Investigate the possible effect of an acute sildenafil dose in asthenozoospermic patients.

**Methods:**

We ran this experimental, descriptive, prospective and non-blinded study in 32 patients diagnosed with asthenozoospermia according to the WHO criteria, after two consecutive semen analyses (samples 1 and 2), and we asked them to provide a new semen sample two weeks after the initial exams (sample 3). One hour prior to the semen collection, we gave the patients 100mg oral sildenafil. Samples 2 and 3 were allowed to liquefy and density gradients centrifugation was performed. We assessed sperm motility by Computer-assisted sperm analysis (CASA).

**Results:**

There were no significant differences between the samples obtained before (sample2) or after sildenafil administration (sample3). However, a single oral 100 mg sildenafil dose in asthenozoospermic patients increase the percentage of motile sperm recovered after density gradient centrifugation.

**Conclusions:**

An acute administration of sildenafil enhances sperm recovery after density gradient centrifugation in asthenozoospermic patients, and it may produce more available mobile sperm to perform intra uterine insemination or to improve the chances of success in this procedure. This approach may be used as an alternative strategy in assisted reproductive programs. Sildenafil was well tolerated and no patient was withdrawn from the study due to adverse events attributed to sildenafil.

## INTRODUCTION

Male infertility comprises any health issue in a man that lowers the chances of his female partner getting pregnant. About 15% of couples can’t get pregnant with unprotected sex (Boivin *et al*., 2007). In about a third of infertility cases, the problem is the man (Agarwal *et al*., 2015). This is most often due to problems with his sperm production or their function. In particular, sperm motility is an important parameter assessed by clinicians when evaluating the male fertility potential. Asthenozoospermia is a decrease in the percentage of motile sperm in a sperm sample, and it is identified by means of a seminogram or semen analysis (Buffone *et al*., 2009). Over the years, several methods have been used to improve sperm motility in those patients with sperm motility defects. This includes the use of pentoxifylline, caffeine, follicular fluids, among others (Drobnis & Nangia, 2017). Most of the time, the goal of this *in vitro* treatment is to increase the percentage of motile forms to improve intrauterine insemination rates, or the number of sperm recovered after sperm separation procedures such as swim up or gradients.

On the other hand, it has been twenty years since the Federal Drug Administration of the United States as the first oral agent to treat erectile dysfunction approved sildenafil citrate (Viagra^®^, Pfizer). Sildenafil acts by blocking phosphodiesterase 5 (PDE5), an enzyme that promotes breakdown of cGMP, which regulates blood flow to the penis (Ramani & Park, 2010) Both, in blood and seminal plasma, Sildenafil citrate concentration reaches a maximum at about 90 minutes after administration (Al-Ghazawi *et al*., 2007) However, the drug remains in micromolar concentrations several hours after its administrations (Al-Ghazawi *et al*., 2007; Gupta *et al*., 2005). This pharmacokinetic feature led us to investigate the possible effects of this drug on sperm function. In this regard, reports on the effects of sildenafil on sperm function are still under debate. Some investigators claim that sildenafil produce no changes (Burger *et al*., 2000; Glenn *et al*., 2009; Purvis *et al*., 2002) while others postulate that this dug promote an improvement in sperm motility (Cuadra *et al*., 2000; Dimitriadis *et al*., 2008).

Although the specific target for sildenafil (PDE5) was not found to be expressed in human sperm in significant levels to be detected, sperm has a wide range of phosphodiesterases involved in cGMP or cAMP, such as PDE1, PDE10, PDE11, PDE8, PDE6 and PDE4 (Buffone *et al*., 2014; Dimitriadis *et al*., 2008) The function of these phosphodiesterases in mammalian spermatozoa remains to be elucidated, but it was reported that Sildenafil’s affinity and selectivity towards all PDEs currently known is much lower than what has been described for PDE5 (Wang *et al*., 2012). Thus, the effects found after sildenafil treatment may not be directly explained by its action on a specific phosphodiesterase and rather, by other mechanisms that involve mitochondrial function and reactive oxygen species (Sousa *et al*., 2014).

Despite that, some studies claim positive effects of Sildenafil on sperm motility, while others report no significant differences. We aimed to investigate the possible effects of an acute Sildenafil dose in asthenozoospermic patients.

## MATERIALS AND METHODS

### Subjects

The study protocol was approved by the Bioethics Committee of the “Dr. José Eleuterio González” University Hospital. These studies comply with the Declaration of Helsinki principles. We provided the patients with written information about the study prior to giving the informed consent. An experimental, descriptive, prospective and non-blinded study was performed in patients consulting for infertility, with ages between 20-45, that were diagnosed with asthenozoospermia according to the World Health Organization (WHO) criteria (WHO, 2010). At least two consecutive sperm analyses with percentage of motility below 40% (A+B) was used as an inclusion criterion. Those patients that were smokers, heavy alcohol consumers, were using any kind of medication or had chronic pathologies were excluded from the study.

Those patients that were diagnosed with asthenozoospermia after two consecutive semen analyses (samples 1 and 2) were asked to provide a new semen sample (sample 3) two weeks after the initial exams. One hour prior to the semen collection, the patients were treated orally with 100 mg of sildenafil.

### Semen analysis and sample preparation

Semen samples were obtained by masturbation after 3-5 days of sex abstinence, and analyzed following WHO recommendations (WHO, 2010). All samples fulfilled semen parameters (total fluid volume, sperm concentration, viability and morphology) according to the WHO normality criteria, with the exception of motility. Samples 2 and 3 were allowed to liquefy for 1h at room temperature. Then, semen aliquots (1 mL) were loaded into a 45 and 90% discontinuous Pure Sperm (Nidacon) gradient (Buffone *et al*., 2012). Density gradients were performed by layering 0.5mL of each pure sperm concentration into a 15mL conical tube. The tube was then centrifuged at 300*g* for 20 min at room temperature. The resulting 90% pellet was then aspirated and transferred to separate tubes. Sperm suspensions were then diluted with HTF (Irvine) medium containing 5% HSA (Human serum albumin) and further centrifuged at 400*g* for 10 min at room temperature. All samples were suspended at 0.5 mL. An aliquot was used to assay sperm concentration and motility.

### Sperm motility evaluation

Sperm motility was assessed by Computer-assisted sperm analysis (CASA), as previously described (Buffone *et al.*, 2006). Aliquots of 5µL of the sperm suspension were placed into a Makler chamber, pre-warmed to 37ºC. CASA analysis was performed using a Hamilton-Thorne digital image analyzer (HTR-IVOS V.12B series 9617; Hamilton-Thorne Research, Beverly, MA). The settings used for the analysis were as follows: frames acquired: 30; frame rate: 60 Hz; minimum contrast: 85; minimum cell size: 4 pixels; straightness threshold: 80%; low path velocity (VAP) cutoff: 5µm/second−1; medium VAP cutoff: 25 µm/second−1; head size, non-motile: 12 pixels; head intensity, non-motile: 130 pixels; static head size: 0.68-2.57 pixels; static head intensity: 0.31-1.25 pixels; and static elongation: 23-100 pixels. The playback function of the HTR was used to accurately identify motile and immotile sperm cells. We have identified as motility type A, sperm that travels 25 µm/sec with linear direction and type B, sperm that travel 5-24 µm/sec without defined direction.

### Statistical analysis

We expressed the data as mean ± standard deviation of the mean (SD). Statistical comparisons were performed by the Student t-test. The calculations were performed with the Libre Office 4.3.2.2 spreadsheet and the statistical analysis with the GraphPad Prism version 4.00 for Windows, GraphPad Software (San Diego, CA, USA). A probability (p) value *p*<0.05 was considered statistically significant.

To determine an appropriate sample size, we ran a power analysis using the STATS software. As a result, a sample size of 32 individuals was necessary to conduct this study (95% confidence).

## RESULTS

A total of 32 male patients with a diagnosis of asthenozoospermia were included in this study. General characteristics of the population such as age, time of infertility and the percentage of individuals who experienced adverse side effects because of sildenafil administration are reported in Table 1. The reported side effects were mild headaches and flushing. However, none of these patients was excluded from this study, since these problems did not affect semen sample quality.

**Table 1:** General features of individuals enrolled in this study

Parameter	Value
Patients (n)	32
Age (years)^†^	32.8 (24-47)
Months of infertility^†^	56.8 (18-144)
Adverse side effects (%)	12.5

† (medium and range).

Table 2 depicts the basic semen parameters. There were no significant differences when the samples obtained before or after sildenafil administration were compared.

**Table 2:** Basic semen parameters before and after sildenafil administration

Parameter	Normal	Before sildenafil ẋ±SD (range)	After sildenafil ẋ±SD (range)	*p*
Volume (mL)	>1.5	2.45±1.4 (0.3-4.8)	2.3±1.1 (0.5-4.3)	NS
Concentration (x10^6^/mL)	≥20	67.3±102.7 (0.5-505)	48.7±38.1 (0.4-150)	NS
Motility A (%)	≥25	11.2±8 (0-24)	14.9±15.6 (0-64)	NS
Motility A+B (%)	≥50	21.4±13 (0-40)	24.6±18.3 (0-70)	NS

ẋ±SD average and standard deviation

Samples were compared using the Student's T-test.

NS Not significantly different

After the initial evaluation, the motile fractions were separated by density gradient centrifugation. As expected, the percentage of motile cells increases after pure sperm separation. However, the percentage of motile cells (A and A+B) was significantly higher in those samples coming from individuals treated with sildenafil, when compared with the same sample obtained two weeks before this treatment (Table 3). The increase in the percentage of mobile cells seen in the sildenafil group was caused by the increase in the number of rapid cells (grade A). There were no significant difference in the percentage of slow cells (B).

**Table 3:** Percentage of sperm motility after density gradient centrifugation with or without sildenafil administration

Parameter	Before sildenafil ẋ±SD (range)	After sildenafil ẋ±SD (range)	*p*
Motility A (%)	16.8 ± 21.4	24.8 ± 23.9	<0.050*
Motility B (%)	7.0 ± 8.4	9.0 ± 8.4	NS
Motility A+B (%)	23.8 ± 22.9	33.8 ± 25.9	<0.050*
Recovery (M/mL)	5.5 ± 6.7	9.9 ± 9.2	<0.050*

Samples were compared using the Student's T-test.

NS Not significantly different

* p<0.050 with statistical significance

When the total number of motile sperm recovered (M/mL) in both conditions was compared, a significantly higher number of sperm was obtained in those samples originated from patients treated with Sildenafil (Table 3).

## DISCUSSION

There are several studies assessing the influence of oral PDE5 inhibitors, such as Sildenafil, on semen parameters. However, the results are controversial and inconsistent (Tan *et al*., 2017). Most seminal PDE5 inhibitor’s effects reported in previous studies were concentrated on sperm motility due to the well-known influence of pentoxifylline and caffeine in this parameter. Because PDE5 hydrolyzes cGMP, inhibition of PDE5 by sildenafil citrate can potentially enhance the effects of cGMP on sperm motility (Dimitriadis *et al*., 2008).

Previous reports demonstrated a significant effect of sildenafil on sperm motility in normospermic and asthenozoospermic patients after incubation of spermatozoa with various doses of sildenafil (Mostafa, 2007; Yang *et al*., 2014) Others reported no *in vitro* effect when using normal donors (Sousa *et al*., 2014). However, there is no conclusive evidence that this effect is due to PDE5 inhibition, and there are no other signaling pathways present in spermatozoa. Because the effect on sperm motility took place 2 hours after the administration of the drug, the sperm epididymal maturation and passage into the vas deferens may be excluded. Thus, this effect appears to be generated in the sperm that are present in the vas ampulla or in the seminal components released by the prostate or the seminal vesicles. Regarding the effects of Sildenafil *in vivo*, several reports have demonstrated contradictory effects (Best reviewed in (Tan *et al*., 2017). Our results show no significant difference in baseline semen parameters before or after Sildenafil. This is in accordance with other reports that did not see major changes in semen volume, sperm concentration, sperm morphology or motility (Aversa *et al*., 2000). However, some authors claimed a mild improvement in some parameters, but only when Sildenafil was used chronically (Dimitriadis *et al*., 2008).

Others have reported a subtle increase in sperm motility after an acute administration (Pomara *et al*., 2007), but in concentrations around 50 mg in contrast to the 100 mg dose that we used in this study. Two groups reported the *in vivo* effect of sildenafil administration on sperm function (Aversa *et al*., 2000; Pomara *et al*., 2007). In addition, it is also reported that the beneficial effects on semen parameters only occurred in infertile individuals, and not in fertile donors (Pomara *et al*., 2007; Rago *et al*., 2012).

In this paper, we report that an acute 100 mg sildenafil administration in asthenozoospermic patients increases the percentage of motile sperm recovered after density gradient centrifugation. This approach may be of great value in those patients with low motility and/or low sperm count, where at the end of the gradient centrifugation, a significant number of sperm is not recovered. Therefore, these patients have no other choice than end up undergoing ICSI or IVF, rather that intrauterine insemination. An acute administration of Sildenafil may produce more available motile sperm to perform intra uterine insemination or to improve the chances of success with this procedure. However, the possible positive effects on the success rate remains to be explored in future studies.

The main limitations of our study are the small number of patients, and the lack of a control group. In our study it was not possible to have a control group; however, it was required that all patients collected two semen samples within three weeks apart, to confirm asthenozoospermia; and two weeks later the patients were treated orally with sildenafil, one hour prior to the collection of a third semen sample. We could considered each patient was his own control.

## CONCLUSIONS

In summary, a single oral Sildenafil treatment increases motile sperm recovery after density gradient centrifugation in asthenozoospermic patients, and may be used as an alternative strategy in assisted reproductive programs. Although chronic administration might yield better effects, this possibility remains to be determined. Large prospective randomized controlled trials are required to confirm these results.
